# Electrical Cardiometry as a Novel Tool for Assessing Systemic Vascular Resistance and Cardiac Function in Obstructive Sleep Apnea

**DOI:** 10.3390/jcm15041530

**Published:** 2026-02-15

**Authors:** Seda Elcim Yildirim, Nurhan Sarioglu, Mustafa Colak, Ibrahim Tanriogen, Tarik Yildirim, Tuncay Kiris, Eyüp Avci

**Affiliations:** 1 Department of Cardiology, School of Medicine, Balikesir University, 10145 Balikesir, Turkey; sedaelcimdurusoy@gmail.com (S.E.Y.); kdrtarik@gmail.com (T.Y.);; 2 Department of Chest Diseases, School of Medicine, Balikesir University, 10145 Balikesir, Turkey; 3 Department of Cardiology, Atatürk Training and Research Hospital, Izmir Katip Çelebi University, 35360 Izmir, Turkey

**Keywords:** electrical cardiometry, OSAS, systemic vascular resistance, cardiac index, hemodynamics, non-invasive monitoring

## Abstract

**Background**: Obstructive sleep apnea syndrome (OSAS) is associated with sympathetic overactivity, intermittent hypoxia, and increased vascular resistance, leading to cardiovascular morbidity. Electrical cardiometry (EC) is a novel, non-invasive technology that continuously measures hemodynamic parameters such as systemic vascular resistance (SVR), systemic vascular resistance index (SVRI), cardiac output (CO), and cardiac index (CI). The aim of this study was to compare SVR and SVRI values, measured by EC, between patients with OSAS and age- and sex-matched healthy controls. **Methods**: In this retrospective case–control study, 70 participants were enrolled, including 33 patients with polysomnography-confirmed OSAS and 37 healthy controls matched for age and sex. All participants underwent standard EC measurement (ICON^®^ Cardiotronics, Osypka Medical, GmbH, Berlin, Germany) under resting, supine conditions. Hemodynamic parameters such as SVR, SVRI, and CI were compared between groups. **Results**: EC revealed significantly higher SVR (1498.7 ± 335.6 vs. 1260.1 ± 251.5 dyn·s·cm^−5^, *p* = 0.013) and SVRI (2969.4 ± 749.1 vs. 2347.4 ± 481.0 dyn·s·cm^−5^·m^2^, *p* < 0.001) in patients with OSAS compared with controls, while CI was significantly lower in the OSAS group (2.6 ± 0.5 vs. 3.2 ± 0.8 L/min/m^2^, *p* < 0.001), indicating increased vascular load and reduced cardiac performance. **Conclusions**: This study is the first to apply EC in OSAS. EC-derived parameters, particularly SVRI and CI, effectively differentiated OSAS patients from healthy subjects, reflecting increased vascular afterload and reduced cardiac performance. These findings suggest that EC is a feasible, non-invasive tool for assessing hemodynamic alterations in OSAS and may have potential for bedside monitoring and future risk stratification studies.

## 1. Introduction

Obstructive sleep apnea syndrome (OSAS) is a highly prevalent disorder characterized by repetitive episodes of upper-airway obstruction during sleep, leading to intermittent hypoxia, intrathoracic pressure swings, and sleep fragmentation. These recurrent cycles of hypoxia–reoxygenation trigger sympathetic activation, oxidative stress, endothelial dysfunction, and systemic inflammation, which together contribute to structural and functional cardiovascular alterations [1,2,3]. A growing body of evidence has demonstrated that OSAS is associated with increased arterial stiffness, elevated blood pressure, and higher cardiovascular morbidity and mortality [4,5,6]. However, the precise hemodynamic changes accompanying OSAS, particularly in terms of systemic vascular resistance (SVR) and cardiac performance, remain incompletely characterized.

In contrast to traditional echocardiography, which offers intermittent structural and functional evaluations, electrical cardiometry (EC) facilitates continuous, non-invasive, beat-to-beat measurement of central hemodynamic parameters, including cardiac output, cardiac index, and systemic vascular resistance [7,8,9]. Moreover, in contrast to tonometric approaches, EC exhibits reduced operator dependency and can be readily used in outpatient and sleep clinic environments [9]. This capacity may provide gradual pathophysiological understanding of autonomic and vascular changes linked to obstructive sleep apnea condition.

EC analyzes thoracic electrical bioimpedance changes during the cardiac cycle and has been validated in diverse clinical settings, including peri-operative hemodynamic monitoring, intensive care, and heart failure [8,9,10]. Although EC has been widely evaluated in surgical and critical-care environments, its potential application in sleep medicine has not been explored. To our knowledge, no published clinical study has utilized EC to assess hemodynamic alterations in patients with OSAS. Given the pathophysiological mechanisms of sympathetic overactivity and vascular remodeling in OSAS, EC-derived parameters—particularly SVR and systemic vascular resistance index (SVRI)—may provide valuable insight into the hemodynamic burden of this disorder. Identifying non-invasive and reproducible markers of vascular load could therefore improve early cardiovascular risk stratification in patients with OSAS.

In this context, the present study aimed to investigate hemodynamic differences between patients with OSAS and healthy controls using EC. Specifically, we sought to determine whether EC-derived SVR and SVRI values differ significantly between the two groups and whether these parameters demonstrate discriminatory capacity in identifying OSAS. We also evaluated the relationship between the apnea–hypopnea index (AHI) and EC-derived hemodynamic measures within the OSAS subgroup. We hypothesized that patients with OSAS would exhibit higher SVR and SVRI and lower cardiac index (CI) compared with healthy controls. By introducing EC technology into this patient population, our study provides novel evidence regarding the feasibility and potential clinical utility of EC for non-invasive assessment of cardiovascular alterations in obstructive sleep apnea.

## 2. Materials and Methods

### 2.1. Study Design and Population

This retrospective case–control study was conducted between January 2025 and August 2025 at the Department of Chest Diseases and Department of Cardiology, School of Medicine, Balikesir University. The study protocol was approved by the local institutional ethics committee (approval date: 30 September 2025; approval no. 2025/7-1) and conducted in accordance with the Declaration of Helsinki.

A total of 70 participants were included in the study: 33 patients with OSAS diagnosed by overnight polysomnography, and 37 age- and sex-matched healthy controls. Inclusion criteria for the OSAS group were age > 18 years, diagnosis of OSAS with an AHI ≥ 5 events/hour, and absence of acute cardiovascular or respiratory instability. Control subjects were recruited from volunteers without symptoms of sleep-disordered breathing and with AHI < 5 events/hour confirmed via polysomnography.

Exclusion criteria included known structural heart disease, moderate-to-severe valvular disease, atrial fibrillation, pulmonary hypertension unrelated to OSAS, active infection, thyroid dysfunction, renal insufficiency (estimated glomerular filtration rate < 60 mL/min/1.73 m^2^), and chronic obstructive pulmonary disease.

### 2.2. Polysomnographic Evaluation

Standard overnight polysomnography (Embla N7000, Medcare Flaga, Reykjavík, Iceland) was performed in all subjects in accordance with the American Academy of Sleep Medicine (AASM) Scoring Manual [11,12]. Polysomnography was performed in all control subjects to exclude obstructive sleep apnea; however, since no OSAS was detected and participants were considered healthy, detailed oxygen saturation parameters were not systematically recorded in this group. Apnea was defined as a complete cessation of airflow for ≥10 s, and hypopnea as a ≥30% reduction in airflow accompanied by ≥3% oxygen desaturation or arousal. The AHI was calculated as the total number of apneas and hypopneas per hour of sleep. According to AHI, OSAS severity was classified as mild (5–15), moderate (15–30), or severe (>30 events/h) [11,12].

### 2.3. Electrical Cardiometry Measurements

All participants underwent EC measurements (ICON^®^ Cardiotronics, Osypka Medical, GmbH, Berlin, Germany) in a quiet room, at rest, in the supine position during daytime hours. Four surface electrodes were placed on the left side of the neck and thorax according to the manufacturer’s guidelines [13,14]. After a 10 min rest period, EC parameters were continuously recorded for at least 5 min and averaged for analysis. EC acquisition is fully automated after electrode placement, and the operator was not formally blinded to group allocation.

The following hemodynamic indices were obtained:

SVR, dyn·s·cm^−5^ or Wood units;

SVRI, dyn·s·cm^−5^·m^2^ or Wood units × m^2^;

Cardiac output (CO; L/min);

CI, L/min/m^2^;

Stroke volume (SV; mL);

Stroke volume index (SVI; mL/m^2^);

Left ventricular ejection time (LVET; ms);

Ambulatory blood pressure monitoring.

Twenty-four-hour ambulatory blood pressure monitoring was initiated on the same day as the EC assessment using a validated oscillometric device (EnviteC PhysioQuant ABPM, model 45-00-0500, EnviteC-Wismar GmbH, Wismar, Germany). Blood pressure measurements were obtained at 20–30 min intervals during the daytime and at 30–60 min intervals during the nighttime, in accordance with standard recommendations. Average morning SBP was calculated as the mean of measurements obtained during the first two hours after awakening. End-night systolic blood pressure was defined as the mean of the last three ambulatory systolic blood pressure measurements obtained during the nocturnal period before awakening.

Venous blood samples were obtained from all participants in the morning of the EC measurement day, after an overnight fast. Standard biochemical analyses, including glucose, lipid profile, and creatinine, were performed using routine automated laboratory methods.

EC has been validated in various clinical contexts, including perioperative monitoring, intensive care, and heart failure [13,14,15]. Because thoracic impedance measurements may be influenced by body habitus, particularly obesity, this factor was considered a potential confounder in the interpretation of EC-derived parameters. EC incorporates body surface area-indexed variables and internal calibration algorithms that may partially mitigate the impact of differences in body composition. SVRI values were obtained using the standard EC-derived body mass indexing. No additional adjustment for body mass index or body surface area was applied beyond the device’s internal calculations. EC-derived parameters were interpreted as estimates of central hemodynamic patterns and group-level differences rather than precise absolute measurements, in accordance with the non-invasive and impedance-based nature of the technique.

### 2.4. Echocardiographic Assessment

Standard transthoracic echocardiography (Vivid E9, GE Healthcare, Chicago, IL, USA) was performed by an experienced cardiologist blinded to group assignment, in accordance with the American Society of Echocardiography (ASE) and European Association of Cardiovascular Imaging (EACVI) recommendations [16]. E/A ratio was assessed using pulsed-wave Doppler at the mitral valve tips according to ASE/EACVI recommendations, and categorical E<A classification was used to avoid potential bias related to beat-to-beat variability. The following parameters were recorded: left ventricular ejection fraction (LVEF, %), left atrial diameter (LA, mm), interventricular septal thickness (IVS, mm), right ventricular diameter (RVd, mm), tricuspid annular plane systolic excursion (TAPSE, mm), and estimated pulmonary artery systolic pressure (PASP, mmHg).

### 2.5. Statistical Analysis

All analyses were performed using R (version 4.3.2) and SPSS (version 26, IBM, Chicago, IL, USA). Data normality was assessed with the Shapiro–Wilk test. Continuous variables were expressed as mean ± standard deviation (SD) or median [interquartile range], and categorical variables as *n* (%). Welch’s t-test for normally distributed variables, Mann–Whitney U test for non-normal data, and χ^2^ or Fisher’s exact test for categorical variables were used. Given the exploratory design and the limited sample size, multivariable adjustment was not performed to avoid model overfitting and unstable estimates. Accordingly, analyses focused on univariable comparisons and exploratory assessment of group separation. Receiver operating characteristic (ROC) curve analysis was performed as a secondary, exploratory analysis to illustrate hemodynamic group separation between patients with obstructive sleep apnea syndrome and controls. ROC results are reported in the Supplementary Materials and were not intended to establish diagnostic accuracy or clinical cut-off values. Statistical significance was defined as *p* < 0.05 (two-tailed).

### 2.6. Sample Size and Power

Given the exploratory nature of this study, formal sample-size calculation was not performed. A post hoc power analysis indicated that the present sample (*n* = 70) provided 80% power (α = 0.05, two-sided) to detect a medium effect size (Cohen’s *d* ≈ 0.7) for differences in SVRI between groups. A post hoc power analysis was performed specifically for SVRI, which demonstrated the strongest group discrimination among EC-derived parameters.

## 3. Results

### 3.1. Baseline Demographic and Clinical Characteristics

A total of 70 participants were analyzed, comprising 33 patients with OSAS and 37 healthy controls. The mean age of groups was similar (53.4 ± 11.0 vs. 51.3 ± 9.9 years, *p* = 0.384, Table 1).

Sex distribution and smoking status were comparable between groups (*p* > 0.1). Comorbidities were significantly more frequent in the OSAS cohort: a hypertension history was more common in OSAS patients compared with controls group (42% vs. 16%, *p* = 0.015, Table 1).

### 3.2. Echocardiographic and Laboratory Findings

**Figure 1:**
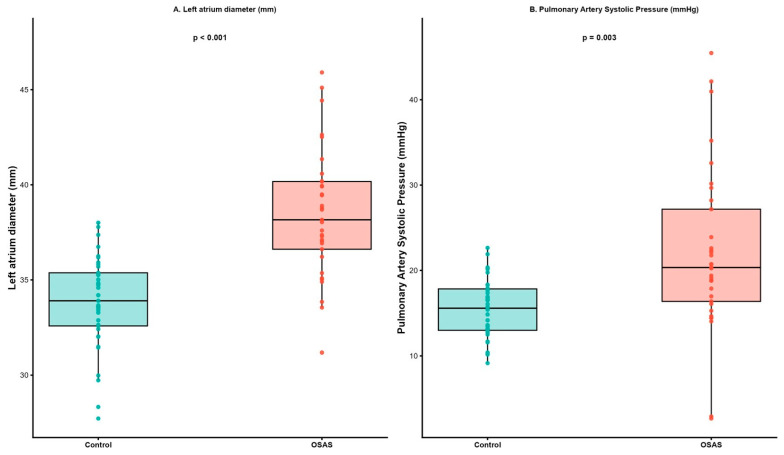
Echocardiographic parameters in the study groups. (**A**) Left atrial diameter. (**B**) Pulmonary artery systolic pressure.

Compared with the control group, patients with OSAS demonstrated significant structural and metabolic alterations. The LA diameter was higher in the OSAS group (37.48 ± 3.47 mm vs. 33.70 ± 2.68 mm, *p* < 0.001, Table 1, Figure 1A), as were the RVd (28.33 ± 3.33 mm vs. 23.68 ± 1.94 mm, *p* < 0.001), aortic diameter (34.18 ± 3.25 mm vs. 32.51 ± 2.27 mm, *p* = 0.017), and LV diastolic diameter (47.00 ± 3.41 mm vs. 44.54 ± 2.59 mm, *p* = 0.001).

Although LVEF values were comparable between groups (63.85 ± 2.41% vs. 64.16 ± 2.15%, *p* = 0.570, Table 2), TR velocity (2.31 ± 0.69 m/s vs. 1.95 ± 0.25 m/s, *p* = 0.007) and PASP (23.09 ± 13.34 mmHg vs. 15.40 ± 4.07 mmHg, *p* = 0.003, Table 2, Figure 1B) were significantly higher in OSAS patients, indicating increased pulmonary pressure. Similarly, IVS thickness was higher in OSAS patients compared with those without (11.61 ± 1.50 mm vs. 10.73 ± 1.41 mm, *p* = 0.014), and the patients with an E<A ratio were more common in the OSAS group (24% vs. 3%, *p* = 0.007, Table 2).

Laboratory results showed higher glucose (113.52 ± 28.22 mg/dL vs. 92.57 ± 9.28 mg/dL, *p* < 0.001), creatinine (0.97 ± 0.20 mg/dL vs. 0.83 ± 0.19 mg/dL, *p* = 0.003, Table 2), and triglyceride levels (220.9 ± 151.5 mg/dL vs. 152.2 ± 75.4 mg/dL, *p* = 0.023) in the OSAS group, while total cholesterol, high-density lipoprotein (HDL) cholesterol, and low-density lipoprotein (LDL) cholesterol were similar between groups (all *p* > 0.05, Table 2).

Ambulatory blood pressure monitoring revealed significantly higher 24 h systolic blood pressure (SBP) [126.2 ± 12.4 mmHg vs. 120.9 ± 6.7 mmHg, *p* = 0.028], 24 h diastolic blood pressure (DBP) [77.3 ± 8.5 mmHg vs. 72.5 ± 6.0 mmHg, *p* = 0.009], nighttime SBP (120.0 ± 14.4 mmHg vs. 111.6 ± 9.5 mmHg, *p* = 0.006), and nighttime DBP (71.4 ± 8.9 mmHg vs. 64.7 ± 7.2 mmHg, *p* < 0.001). Additionally, average-morning SBP was significantly higher in OSAS patients than in the control groups (128.2 ± 14.2 vs. 118.8 ± 10.3, *p* = 0.002, Table 2).

### 3.3. Electrical Cardiometry Findings

**Figure 2:**
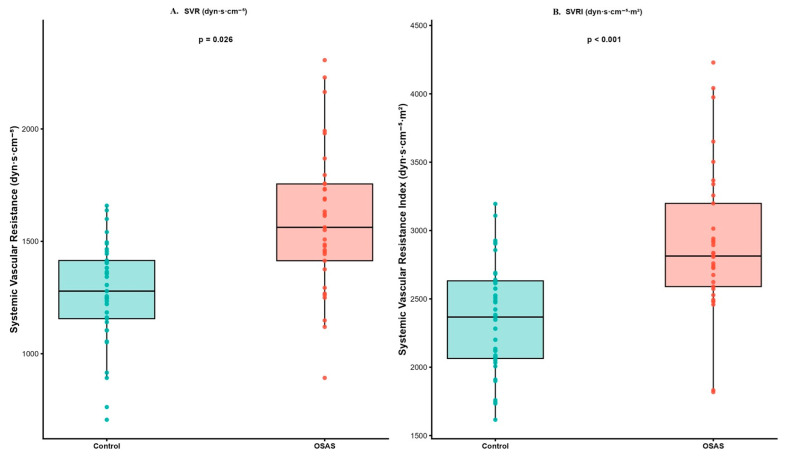
Systemic vascular resistance parameters derived from electrical cardiometry in the study groups. (**A**) Systemic vascular resistance (SVR). (**B**) Systemic vascular resistance index (SVRI).

**Figure 3:**
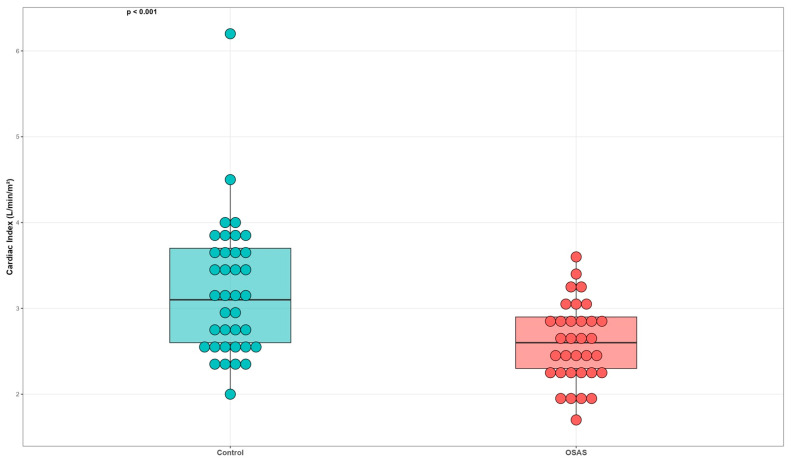
Cardiac index (CI) values in the groups. Individual data points represent discrete cardiac index values obtained by electrical cardiometry. Minimal horizontal jitter was applied for visualization purposes only and does not indicate additional measurement variability.

When EC parameters were compared between the OSAS and control groups, significant hemodynamic differences were observed (Table 3). The SVR and SVRI were markedly higher in patients with OSAS compared with controls (1498.7 ± 335.6 vs. 1260.1 ± 251.5 dyn·s·cm^−5^, *p* = 0.013; 2969.4 ± 749.1 vs. 2347.4 ± 481.0 dyn·s·cm^−5^·m^2^, *p* < 0.001, respectively, Figure 2). Conversely, CI was significantly lower in the OSAS group (2.6 ± 0.5 vs. 3.2 ± 0.8 L/min/m^2^, *p* < 0.001, Figure 3), indicating a relative reduction in cardiac output per body surface area. Although CO tended to be lower in OSAS patients (5.4 ± 1.0 vs. 5.8 ± 1.2 L/min), this difference did not reach statistical significance (*p* = 0.115). The lower CI observed in the OSAS group should be interpreted in the context of body surface area indexing, as similar absolute stroke volume values translated into lower indexed cardiac performance due to higher body mass in OSAS patients.

No significant intergroup differences were found in SV (65.1 ± 8.2 vs. 66.4 ± 11.6 mL, *p* = 0.597) or LVET (273.9 ± 31.7 vs. 263.1 ± 25.9 ms, *p* = 0.121). However, SVI was lower in patients with OSAS than those without OSAS (1.93 ± 0.48 vs. 2.73 ± 0.52, *p* < 0.001, Table 3). Exploratory ROC analyses evaluating group separation are provided in the Supplementary Materials (Figure S1).

## 4. Discussion

This study is the first, to our knowledge, to investigate hemodynamic alterations in patients with OSAS using EC. Our findings demonstrate that patients with OSAS exhibit significantly higher SVR and SVRI and lower CI compared with age- and sex-matched healthy controls, despite preserved total CO. Additionally, echocardiographic measurements revealed increased left-atrial size, right-ventricular dimensions, and pulmonary pressures, supporting the concept of early cardiovascular remodeling in OSAS.

The observed increase in SVR and SVRI reflects the elevated systemic vascular tone associated with chronic sympathetic activation in OSAS. Recurrent episodes of hypoxia and arousal during sleep lead to surges in catecholamine release and sustained endothelial dysfunction, promoting vasoconstriction and impaired nitric oxide bioavailability [17,18,19,20]. Previous studies using invasive or Doppler-based methods have similarly reported elevated peripheral vascular resistance in OSAS, particularly in those with moderate-to-severe disease [21,22]. Our results extend these observations by showing that these alterations can be detected non-invasively through EC, even in stable outpatients.

The finding of reduced CI but preserved stroke volume and ejection fraction suggests that cardiac output is maintained at the expense of increased afterload and compensatory tachycardia in OSAS. Notably, SV did not differ significantly between groups, whereas CI was lower in patients with OSAS. This discrepancy is largely explained by body surface area indexing, as cardiac index represents CO normalized to body surface area. Given the higher body mass index in the OSAS group, similar absolute SV translated into a lower-indexed cardiac performance, highlighting the importance of considering body size when interpreting EC-derived indices. Increased LV end-diastolic diameter, and LA enlargement in our cohort are consistent with diastolic dysfunction and volume overload, both commonly reported in OSAS patients [23,24]. Similarly, elevated PASP and RVd indicate early pulmonary hypertension due to recurrent hypoxic vasoconstriction and intermittent pressure overload [25,26]. Taken together, these findings support the hypothesis that OSAS induces a global hemodynamic strain affecting both systemic and pulmonary circulations.

EC is an impedance-based technique that estimates SV and related indices from thoracic conductivity changes during the cardiac cycle. Compared with classical impedance cardiography, EC incorporates improved signal filtering and algorithmic calibration, enabling higher reliability in clinical settings [7,9,10]. Our results highlight the potential of EC as a rapid, reproducible, and non-invasive modality for hemodynamic profiling in OSAS. Importantly, EC-derived SVRI demonstrated good discriminatory performance for identifying OSAS comparable to more complex echocardiographic indices. This suggests that EC could serve as a valuable bedside screening tool for vascular load assessment and early cardiovascular risk stratification in sleep-disordered breathing. Although EC offers clear advantages in terms of non-invasiveness, ease of use, and feasibility for repeated measurements, its absolute accuracy may be lower than that of invasive reference techniques such as thermodilution. Previous validation studies have shown moderate agreement between EC and thermodilution-derived cardiac output, particularly under stable hemodynamic conditions [7,8]. Therefore, EC-derived parameters in the present study should be interpreted as reflective of relative hemodynamic patterns and group-level differences rather than precise absolute values.

From a clinical standpoint, the hemodynamic profile revealed by EC aligns with the known neurohumoral and vascular consequences of OSAS. Increased SVR and reduced CI reflect a maladaptive hemodynamic pattern that contributes to hypertension and left-ventricular remodeling. Early identification of such abnormalities may allow clinicians to target blood pressure control, endothelial function, and sympathetic modulation before irreversible cardiac damage develops. Furthermore, EC could be incorporated into longitudinal follow-up protocols to evaluate the impact of continuous positive airway pressure (CPAP) therapy on vascular resistance and cardiac function.

In normotensive controls, preserved baroreflex sensitivity and vascular compliance may result in compensatory vasodilation despite higher systolic pressures, leading to lower calculated SVR and SVRI values. In contrast, OSAS is characterized by impaired autonomic modulation and endothelial dysfunction, resulting in a more fixed and elevated vascular tone [17,18].

Most previous studies evaluating OSAS-related hemodynamics have relied on echocardiography or tonometric measurements, which are operator-dependent and not suitable for continuous monitoring [27,28]. Our data suggest that EC can provide similar physiological insights with minimal patient discomfort and at a lower cost. Although absolute accuracy compared to thermodilution remains debated, EC’s reproducibility and trending ability make it highly attractive for research and outpatient care.

Several limitations should be acknowledged. First, the sample size was modest, and this study was designed to be exploratory; thus, findings should be confirmed in larger cohorts. Second, EC-derived values are influenced by body composition and thoracic impedance, which may differ between obese OSAS patients and lean controls. Third, although not evaluated in the present study, EC may represent a promising hypothesis-generating tool for longitudinal assessment of hemodynamic changes during CPAP therapy. Future prospective studies are required to determine whether EC-derived parameters are sensitive to treatment-related cardiovascular adaptations in patients with OSAS. Fourth, body mass index and the prevalence of hypertension were higher in the OSAS group. Also, an important limitation of this study is the lack of information regarding the duration of OSAS prior to diagnosis, as well as the absence of formal matching for blood pressure and antihypertensive treatment status, which may introduce residual confounding when interpreting electrical cardiometry-derived hemodynamic parameters. Fifth, in addition to anthropometric and clinical differences, laboratory parameters such as glucose, triglycerides, and creatinine levels were higher in the OSAS group, suggesting a broader cardiometabolic phenotype. These metabolic alterations are known to affect vascular tone, endothelial function, and systemic vascular resistance and may have influenced EC-derived hemodynamic measurements. Sixth, although SVRI is indexed to body mass, residual confounding related to obesity cannot be fully excluded, as no additional BMI-adjusted analyses were performed. Seventh, multivariable adjustment was not performed, because of the modest sample size and exploratory design. Residual confounding related to differences in body mass index and blood pressure cannot be excluded, and EC parameters were not analyzed according to OSAS severity; thus, potential dose–response relationships could not be evaluated. Eighth, although polysomnography was performed in the control group to exclude OSAS, detailed oxygen saturation and respiratory parameters were not systematically recorded, which may limit deeper comparisons of nocturnal hypoxemia between groups, and EC-derived measurements represent non-invasive estimates rather than invasive gold-standard values. Therefore, the present findings should be interpreted as reflecting relative hemodynamic patterns rather than exact absolute values. Finally, our cross-sectional design precludes establishing causality between OSAS severity and vascular resistance.

In conclusion, our study provides novel evidence that EC can detect significant hemodynamic alterations in patients with obstructive sleep apnea. OSAS is associated with increased systemic vascular resistance and decreased cardiac index, reflecting an adverse hemodynamic profile linked to sympathetic overactivation and vascular remodeling. These findings support the potential utility of EC as a non-invasive, practical tool for cardiovascular risk evaluation and monitoring in sleep medicine. Importantly, EC should be viewed as an adjunctive and exploratory tool for non-invasive assessment of hemodynamic burden in OSAS, rather than as a standalone diagnostic modality. While echocardiography and ambulatory blood pressure monitoring provide structural and peripheral pressure information, EC offers direct insight into systemic vascular load, which is not obtainable from these modalities alone. Given the cross-sectional design, these findings should be interpreted as associations and do not imply causal relationships.

## Figures and Tables

**Table 1:** Demographic and clinical variables.

Variables	Control (*n* = 37)	OSAS (*n* = 33)	*p*-Value
Age (years)	51.3 ± 9.9	53.4 ± 11.0	0.384
Height (cm)	170.7 ± 9.5	167.7 ± 7.8	0.162
Weight (kg)	70.9 ± 9.2	97.9 ± 17.2	<0.001
Body mass index (kg/m^2^)	24.3 ± 1.8	35.2 ± 7.4	<0.001
Male gender *n* (%)	18 (49)	20 (61)	0.316
Smoking *n* (%)	13 (37)	13 (39)	0.849
Hypertension *n* (%)	6 (16)	14 (42)	0.015
Diabetes mellitus *n* (%)	1 (3)	4 (12)	0.127

Abbreviations: OSAS, obstructive sleep apnea syndrome.

**Table 2:** Comparison of echocardiographic and laboratory parameters between OSAS and control groups.

Variable	Control (*n* = 37)	OSAS (*n* = 33)	*p*-Value
Heart rate (bpm)	77.38 ± 8.48	77.36 ± 10.35	0.995
LV diastolic diameter (mm)	44.54 ± 2.59	47.00 ± 3.41	0.001
Aortic diameter (mm)	32.51 ± 2.27	34.18 ± 3.25	0.017
Left atrial diameter (mm)	33.70 ± 2.68	37.48 ± 3.47	<0.001
RV diameter (mm)	23.68 ± 1.94	28.33 ± 3.33	<0.001
TAPSE (mm)	20.46 ± 2.01	19.88 ± 2.21	0.256
TR velocity (m/s)	1.95 ± 0.25	2.31 ± 0.69	0.007
LVEF (%)	64.16 ± 2.15	63.85 ± 2.41	0.570
PASP (mmHg)	15.40 ± 4.07	23.09 ± 13.34	0.003
IVS thickness (mm)	10.73 ± 1.41	11.61 ± 1.50	0.014
Patients with E < A, *n* (%)	1 (3)	8 (24)	0.007
Glucose (mg/dL)	92.57 ± 9.28	113.52 ± 28.22	<0.001
Creatinine (mg/dL)	0.83 ± 0.19	0.97 ± 0.20	0.003
Total cholesterol (mg/dL)	188.6 ± 46.3	197.1 ± 40.8	0.416
Triglycerides (mg/dL)	152.2 ± 75.4	220.9 ± 151.5	0.023
HDL-cholesterol (mg/dL)	47.0 ± 12.3	42.6 ± 10.3	0.109
LDL-cholesterol (mg/dL)	119.1 ± 31.4	116.1 ± 28.9	0.690
WBC (×10^3^/µL)	7.6 ± 2.2	8.7 ± 2.3	0.072
Hemoglobin (g/dL)	13.78 ± 1.66	14.30 ± 1.28	0.144
Platelet (×10^3^/µL)	287.3 ± 73.0	286.4 ± 78.4	0.963
24 h SBP (mmHg)	120.9 ± 6.7	126.2 ± 12.4	0.028
24 h DBP (mmHg)	72.5 ± 6.0	77.3 ± 8.5	0.009
Daytime SBP (mmHg)	123.3 ± 6.9	128.2 ± 12.7	0.055
Daytime DBP (mmHg)	74.5 ± 6.3	78.9 ± 8.9	0.020
Nighttime SBP (mmHg)	111.6 ± 9.5	120.0 ± 14.4	0.006
Nighttime DBP (mmHg)	64.7 ± 7.2	71.4 ± 8.9	<0.001
End-night SBP (mmHg)	93.5 ± 9.9	102.8 ± 11.4	<0.001
Average morning SBP (mmHg)	118.8 ± 10.3	128.2 ± 14.2	0.002

Abbreviations: DBP, diastolic blood pressure; LVEF, left ventricular ejection fraction; HDL, high-density lipoprotein; IVS, interventricular septum; LDL, low-density lipoprotein; LV, left ventricle; PASP, pulmonary artery systolic pressure; RV, right ventricle; SBP, systolic blood pressure; TAPSE, tricuspid annular plane systolic excursion; TR, tricuspid regurgitation; WBC, white blood cell count; OSAS, obstructive sleep apnea syndrome.

**Table 3:** Electrical cardiometry parameters between groups.

Variables	Control (*n* = 37)	OSAS (*n* = 33)	*p*-Value
SVR (dyn·s·cm^−5^)	1260.1 ± 251.5	1498.7 ± 335.6	0.013
SVR (Wood units)	15.8 ± 3.1	18.7 ± 4.2	0.013
SVRI (dyn·s·cm^−5^·m^2^)	2347.4 ± 481.0	2969.4 ± 749.1	<0.001
SVRI (Wood units × m^2^)	29. 3 ± 6.0	37.1 ± 9.4	<0.001
CO (L/min)	5.8 ± 1.2	5.4 ± 1.0	0.115
CI (L/min/m^2^)	3.2 ± 0.8	2.6 ± 0.5	<0.001
Stroke volume (mL)	66.4 ± 11.6	65.1 ± 8.2	0.597
Stroke volume index (mL/m^2^)	2.73 ± 0.52	1.93 ± 0.48	<0.001
LVET (ms)	263.1 ± 25.9	273.9 ± 31.7	0.121

Abbreviations: SVR, systemic vascular resistance; SVRI, systemic vascular resistance index; CO, cardiac output; CI, cardiac index; LVET, left ventricular ejection time; OSAS, obstructive sleep apnea syndrome.

## Data Availability

The data presented in this study are available upon request from the corresponding author due to privacy.

## Supplementary Materials

The following supporting information can be downloaded at: https://www.mdpi.com/article/10.3390/jcm15041530/s1, Figure S1. The area under the curve (AUC) with 95% confidence interval of SVR, SVRI, and CI.

## References

[1] Garvey J.F., Taylor C.J., McNicholas W.T. (2009). Cardiovascular disease in obstructive sleep apnoea. Eur. Respir. J..

[2] Gozal D., Kheirandish-Gozal L. (2008). Cardiovascular morbidity in obstructive sleep apnea: Oxidative stress, inflammation, and much more. Am. J. Respir. Crit. Care Med..

[3] Turnbull C.D. (2018). Intermittent hypoxia, cardiovascular disease and obstructive sleep apnoea. J. Thorac. Dis..

[4] Dinç Y., Demir A.B. (2022). Obstructive sleep apnea syndrome and cardiovascular diseases; the role of hypertension. J. Turk. Sleep Med..

[5] Dewan N.A., Nieto F.J., Somers V.K. (2015). Intermittent hypoxemia and OSA: Implications for comorbidities. Chest.

[6] Müller M.B., Stihl C., Schmid A., Hirschberger S., Mitsigiorgi R., Holzer M., Patscheider M., Weiss B.G., Reichel C., Hübner M. (2023). A novel OSA-related model of intermittent hypoxia in endothelial cells under flow reveals pronounced inflammatory pathway activation. Front. Physiol..

[7] Sanders M., Servaas S., Slagt C. (2020). Accuracy and precision of non-invasive cardiac output monitoring by electrical cardiometry: A systematic review and meta-analysis. J. Clin. Monit. Comput..

[8] Greiwe G., Saad R., Hapfelmeier A., Neumann N., Tariparast P., Saugel B., Flick M. (2025). Electrical cardiometry for non-invasive cardiac output monitoring: A method comparison study in patients after coronary artery bypass graft surgery. J. Clin. Monit. Comput..

[9] Yassen K.A., Aljumaiy W., Alherz I., AlMudayris L.A., AlBunyan S.A., AlSubaie R.S., Alniniya F., Saleh S. (2025). Non-Invasive Cardiac Output Monitoring with Electrical Cardiometry During Laparoscopic Cholecystectomy Surgery, a Cross-Sectional Study. J. Clin. Med..

[10] Rao S.S., Lalitha A.V., Reddy M., Ghosh S. (2021). Electrocardiometry for Hemodynamic Categorization and Assessment of Fluid Responsiveness in Pediatric Septic Shock: A Pilot Observational Study. Indian J. Crit. Care Med..

[11] Berry R.B., Brooks R., Gamaldo C., Harding S.M., Lloyd R.M., Quan S.F., Troester M.T., Vaughn B.V. (2020). The AASM Manual for the Scoring of Sleep and Associated Events: Rules, Terminology and Technical Specifications.

[12] Mehra R., Auckley D.H., Johnson K.G., Billings M.E., Carandang G., Falck-Ytter Y., Khayat R.N., Mustafa R.A., Pena-Orbea C., Sahni A.S. (2025). Evaluation and management of obstructive sleep apnea in adults hospitalized for medical care: An American Academy of Sleep Medicine clinical practice guideline. J. Clin. Sleep Med..

[13] Bernstein D.P. (1986). A new stroke volume equation for thoracic electrical bioimpedance: Theory and rationale. Crit. Care Med..

[14] Cox P.B.W., den Ouden A.M., Theunissen M., Montenij L.J., Kessels A.G.H., Lancé M.D., Buhre W.F.F.A., Marcus M.A.E. (2017). Accuracy, Precision, and Trending Ability of Electrical Cardiometry Cardiac Index versus Continuous Pulmonary Artery Thermodilution Method: A Prospective, Observational Study. Biomed Res Int..

[15] Samir M., Mostafa F.A., Sobhy R., El-Sisi A., Tantawy A.E., Sakr H.M., Afifi A. (2024). Electrical cardiometry significance in postoperative cardiac ICU monitoring. Egypt. J. Anaesth..

[16] Lang R.M., Badano L.P., Mor-Avi V., Afilalo J., Armstrong A., Ernande L., Flachskampf F.A., Foster E., Goldstein S.A., Kuznetsova T. (2015). Recommendations for cardiac chamber quantification by echocardiography in adults. J. Am. Soc. Echocardiogr..

[17] Somers V.K., Dyken M.E., Clary M.P., Abboud F.M. (1995). Sympathetic neural mechanisms in obstructive sleep apnea. J. Clin. Investig..

[18] Lavie L. (2012). Oxidative stress and endothelial dysfunction in obstructive sleep apnea. Front. Physiol..

[19] Tamisier R., Pépin J.L., Rémy J., Baguet J.P., Taylor J.A., Weiss J.W., Lévy P. (2015). 14-day measurement of hemodynamic response to intermittent hypoxia. Eur. Respir. J..

[20] Jelic S., Lederer D.J., Adams T., Padeletti M., Colombo P.C., Factor P.H., Le Jemtel T.H. (2010). Vascular inflammation in obesity and sleep apnea. Circulation.

[21] Tsioufis C., Thomopoulos K., Dimitriadis K., Amfilochiou A., Tousoulis D., Alchanatis M., Stefanadis C., Kallikazaros I. (2007). The incremental effect of obstructive sleep apnoea syndrome on arterial stiffness in newly diagnosed essential hypertensive subjects. J. Hypertens..

[22] Drager L.F., Bortolotto L.A., Maki-Nunes C., Trombetta I.C., Alves M.J., Fraga R.F., Negrão C.E., Krieger E.M., Lorenzi-Filho G. (2010). The incremental role of obstructive sleep apnoea on markers of atherosclerosis in patients with metabolic syndrome. Atherosclerosis.

[23] Holtstrand Hjälm H., Fu M., Hansson P.-O., Zhong Y., Caidahl K., Mandalenakis Z., Morales D., Ergatoudes C., Rosengren A., Grote L. (2018). Association between left atrial enlargement and obstructive sleep apnea in a general population of 71-year-old men. J. Sleep Res..

[24] Imai Y., Tanaka N., Usui Y., Takahashi N., Kurohane S., Takei Y., Takata Y., Yamashina A. (2015). Severe obstructive sleep apnea increases left atrial volume independently of left ventricular diastolic impairment. Sleep Breath..

[25] Arias M.A., García-Río F., Alonso-Fernández A., Martínez I., Villamor J. (2006). Pulmonary hypertension in obstructive sleep apnoea: Effects of continuous positive airway pressure. Eur. Heart J..

[26] Marrone O., Bonsignore M.R. (2011). Pulmonary hemodynamics in OSA: Evidence and controversies. Chest.

[27] Parati G., Lombardi C., Hedner J., Bonsignore M.R., Grote L., Tkacova R., Levy P., Riha R., Bassetti C., Narkiewicz K. (2012). Position paper on the management of patients with obstructive sleep apnea and hypertension: Joint recommendations by the European Society of Hypertension, by the European Respiratory Society and by the members of European COST (COoperation in Scientific and Technological research) ACTION B26 on obstructive sleep apnea. J. Hypertens..

[28] Lorne E., Mahjoub Y., Guinot P.G. (2015). Continuous non-invasive hemodynamic monitoring: State of the art. Ann. Intensive Care.

