# Supplementary figures and images for: Electrical Cardiometry as a Novel Tool for Assessing Systemic Vascular Resistance and Cardiac Function in Obstructive Sleep Apnea
Source: J Clin Med. 2026 Feb 15;15(4):1530. doi: 10.3390/jcm15041530 (PMC12942121; doi:10.3390/jcm15041530)

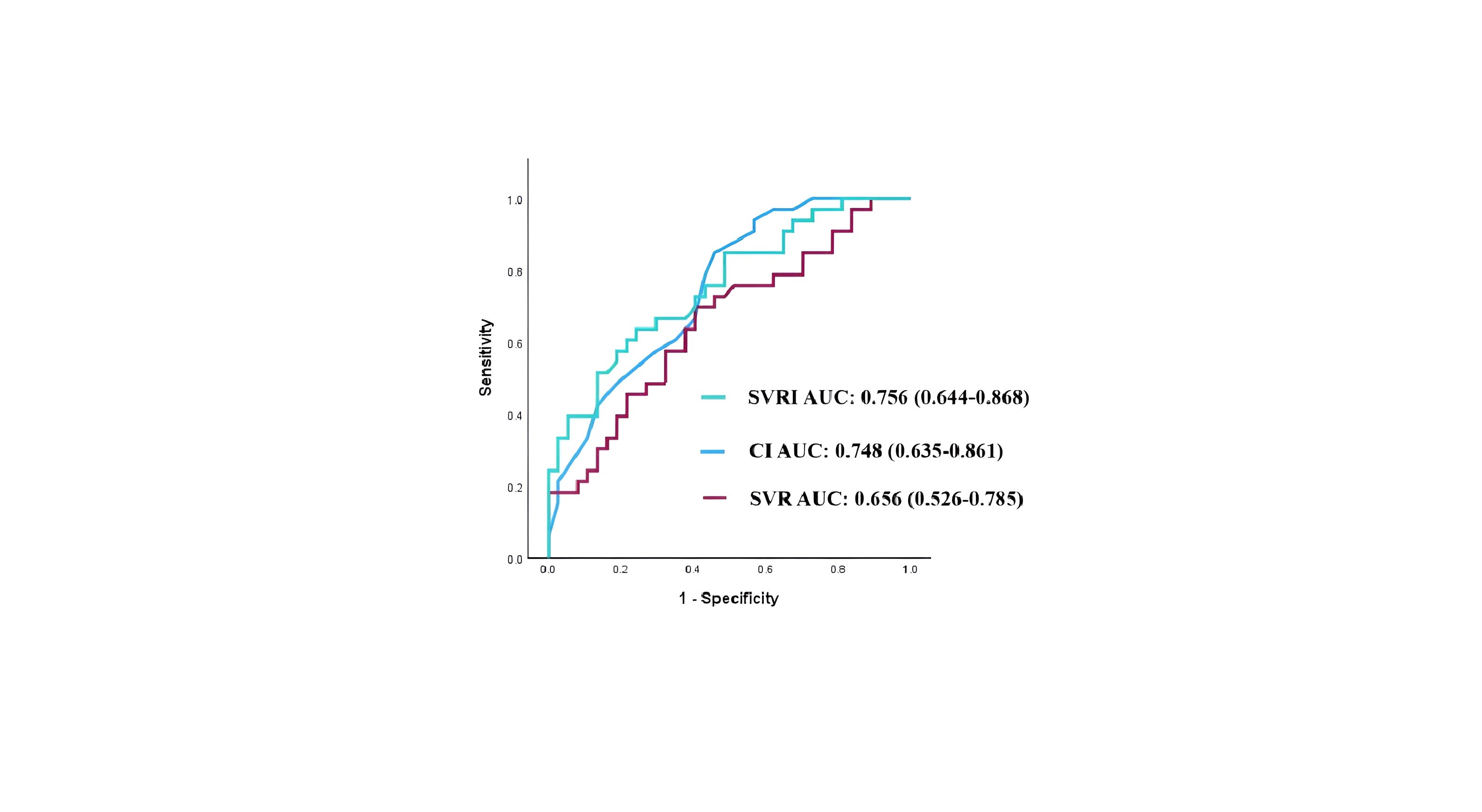

Supplement: Supplementary file 1 [file jcm-15-01530-s001.zip › jcm-4135580-supplementary.jpeg]
